# *AutoPath*: Image-Specific Inference for 3D Segmentation

**DOI:** 10.3389/fnbot.2020.00049

**Published:** 2020-07-24

**Authors:** Dong Sun, Yi Wang, Dong Ni, Tianfu Wang

**Affiliations:** National-Regional Key Technology Engineering Laboratory for Medical Ultrasound, Guangdong Provincial Key Laboratory of Biomedical Measurements and Ultrasound Imaging, School of Biomedical Engineering, Health Science Center, Shenzhen University, Shenzhen, China

**Keywords:** segmentation, 3D residual networks, reinforcement learning, policy network, image-specific inference

## Abstract

In recent years, deep convolutional neural networks (CNNs) has made great achievements in the field of medical image segmentation, among which residual structure plays a significant role in the rapid development of CNN-based segmentation. However, the 3D residual networks inevitably bring a huge computational burden to machines for network inference, thus limiting their usages for many real clinical applications. To tackle this issue, we propose *AutoPath*, an image-specific inference approach for more efficient 3D segmentations. The proposed *AutoPath* dynamically selects enabled residual blocks regarding different input images during inference, thus effectively reducing total computation without degrading segmentation performance. To achieve this, a policy network is trained using reinforcement learning, by employing the rewards of using a minimal set of residual blocks and meanwhile maintaining accurate segmentation. Experimental results on liver CT dataset show that our approach not only provides efficient inference procedure but also attains satisfactory segmentation performance.

## 1. Introduction

Automated segmentation is useful to assist doctors in disease diagnosis and surgical/treatment planning. Since deep learning (LeCun et al., 2015) has utilized widely, medical image segmentation has made great progresses. Various architectures of deep convolutional neural networks (CNNs) have been proposed and successfully introduced to many segmentation applications. Among various architectures, the residual structures in ResNet (He et al., 2016) play an important role in the rapid development of CNN-based segmentation. The backbone which contains the residual blocks has become essential support for many segmentation models, such as DeepLab V3 (Chen et al., 2017), HD-Net (Jia et al., 2019), Res-UNet (Xiao et al., 2018), and so on. Despite the superior performance of residual blocks, these structures inevitably bring a huge computational burden for network inference. This leads to the difficulty of introducing deep models (such as 3D ResNet-50/101) in clinical practice.

Recently, some strategies for model compression have been devoted to tackling the problem of large computation, among which the network pruning approaches have been extensively investigated (Li et al., 2016; He et al., 2017, 2018; Liu et al., 2017; Luo et al., 2017). In addition, other researches are focusing on lightweight network architectures (Howard et al., 2017; Ma et al., 2018; Mehta et al., 2018). However, all aforementioned methods including pruning, knowledge distillation, and lightweight structures all require network retraining and hyper-parameters retuning, which may consume plenty of extra time.

**Figure 1:**
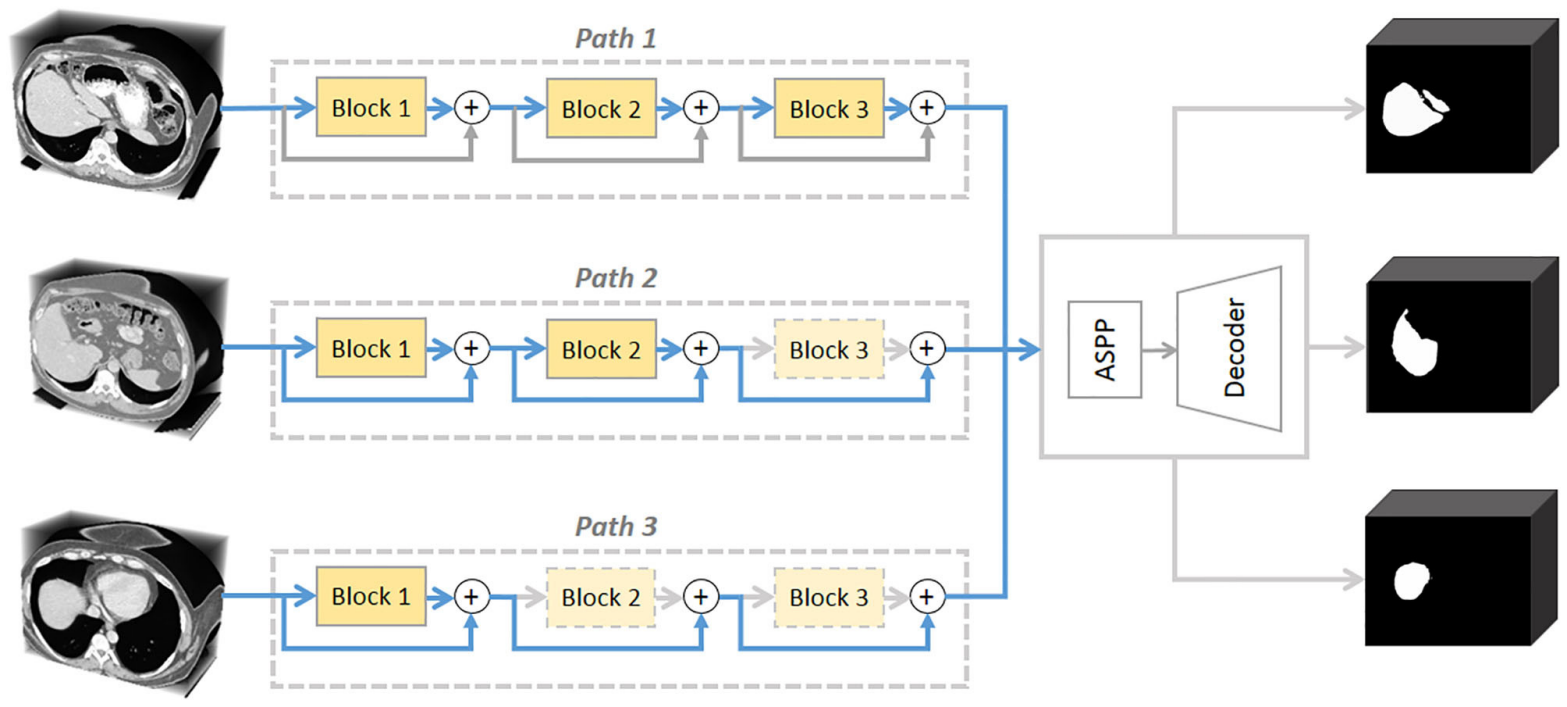
A conceptual illustration of the proposed *AutoPath*. The motivation is to dynamically distributing computation across a ResNet. *AutoPath* selectively drops unnecessary residual blocks for image-specific inference. Such inference simultaneously achieves efficiency and accuracy.

This paper explores the problem of dynamically distributing computation across all residual blocks in a trained ResNet for image-specific segmentation inference (see Figure 1). Relevant studies have been investigated in classification tasks. Teerapittayanon et al. (2016) developed BranchyNet to conduct fast inference via early exiting from deep neural networks. Graves (2016) devised an adaptive computation time (ACT) approach for recurrent neural network (RNN), by designing a halting unit whose activation indicates the termination probability of computations. Huang et al. (2016) proposed stochastic depth for deep networks, a training strategy that enables the seemingly contradictory setup to train short networks and use deep networks at test time. Veit et al. (2016) proposed a description of residual networks in classification showing that residual blocks can be seen as a collection of many paths and they do not strongly depend on each other thus can be selectively dropped. However, there are two obvious differences between segmentation and classification tasks: (1) The neural networks for classification often take a short approach, such as identifying a car by its shadow. Segmentation is classifying each pixel, thus the neural networks cannot be lazy; (2) Classification can work with local features, but segmentation needs to take global information (such as shape priors) into consideration.

In this study, we propose *AutoPath*, an image-specific strategy to design the inference path that uses minimal residual blocks but still preserves satisfactory segmentation accuracy. Specifically, a policy network is trained using reinforcement learning, by using the rewards of involving a minimal set of residual blocks and meanwhile maintaining accurate segmentation. To the best of our knowledge, this is the first investigation of the dynamic inference path for 3D segmentation using residual networks. Experimental results demonstrate that our strategy not only provides efficient inference procedure but also attains satisfactory segmentation accuracy.

## 2. Methods

**Figure 2:**
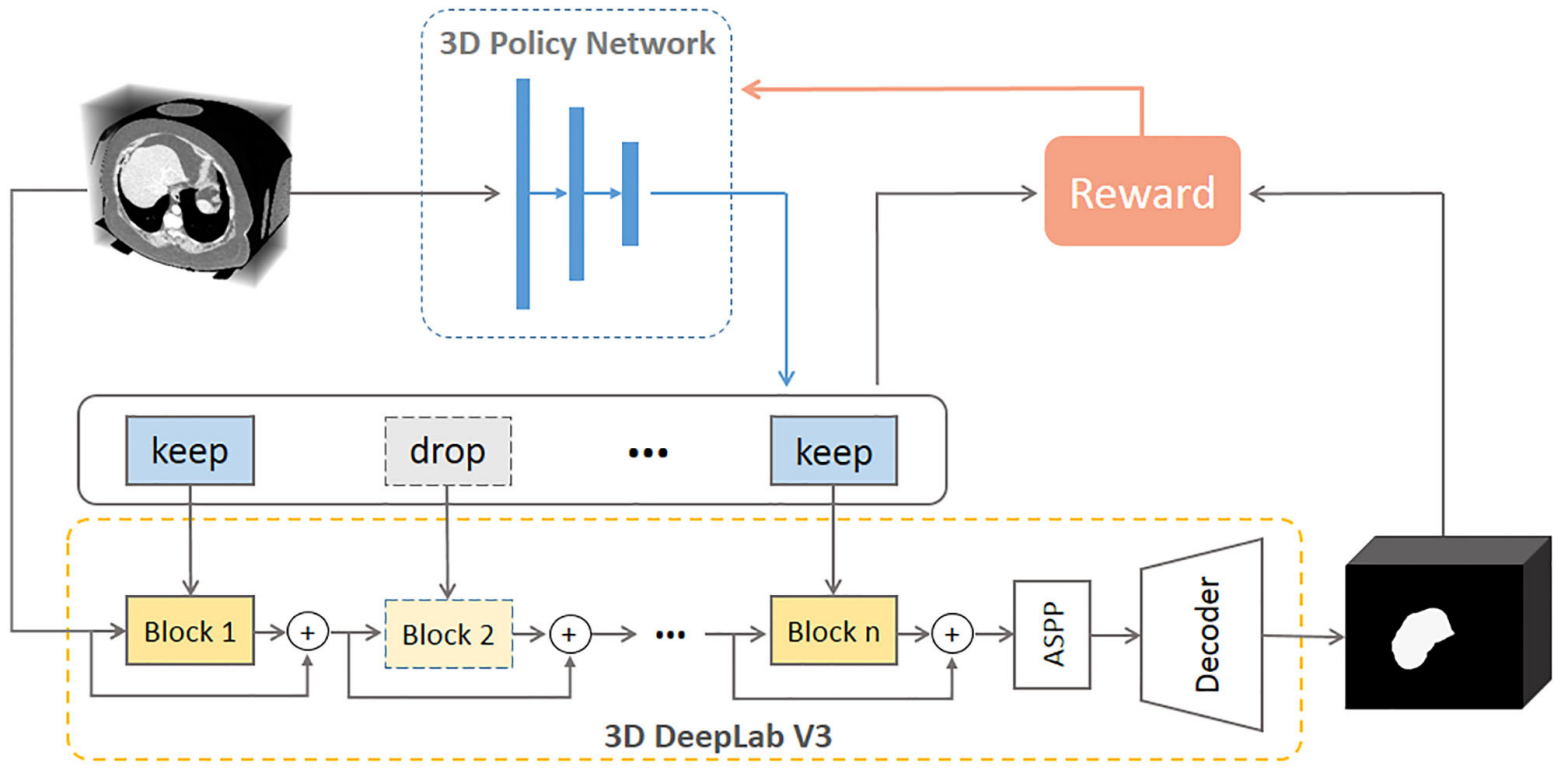
Illustration of the proposed network. The 3D policy network is trained using reinforcement learning with the reward as both segmentation performance and residual block usage. The 3D DeepLab V3 leverages the decisions made by the policy network to keep or drop corresponding residual blocks then outputs the segmentation prediction for a given 3D volume. ASPP: atrous spatial pyramid pooling.

Figure 2 illustrates the proposed framework. Given a new 3D volume, the policy network outputs “keep” or “drop” decisions for each residual block in the pretrained 3D DeepLab V3 network. Such image-specific inference path is then used for the segmentation prediction. The policy network is trained using reinforcement learning by rewarding accurate segmentation with minimal involved blocks.

### 2.1. 3D Residual Backbone

We implement a 3D DeepLab V3 based on the original DeepLab V3 (Chen et al., 2017), and further set its backbone to 3D ResNet according to ResNet (He et al., 2016) (the classification head is removed). We then specifically modify the 3D residual blocks to achieve better baseline results. Optimized 3D residual blocks assign the stride of each convolutional layer along *z* axis to 1 to constrain downsampling along the slice direction. In addition, we replace the straightforward upsampling operations after atrous spatial pyramid pooling (ASPP) with a decoder consisting of a series of convolutions according to Chen et al. (2019).

### 2.2. *AutoPath* Strategy

Backbone, as an indispensable part of segmentation networks, occupies most of the memory and calculation. Generally, the popular residual backbone consists of multiple repeated residual blocks. We regard each block as an independent decision unit by assuming that different blocks do not share strong dependencies in segmentation. Then as shown in Figure 2, we introduce reinforcement learning to train a policy network to intelligently conduct block dropping and explore the inference path that generates accurate segmentation with fewer blocks.

#### 2.2.1. 3D Policy Network

Considering the actions for residual blocks can only be “keep” or “drop,” we define the policy for block dropping as a Bernoulli distribution (Wu et al., 2018):

(1) $$\pi_{W}(a|x)=\prod_{n=1}^{N}p_{n}^{a_{n}}{\left(1-p_{n}\right)}^{1-a_{n}},$$

where **x** denotes the input 3D image and *N* is the total number of residual blocks in the pretrained 3D DeepLab V3 network. **W** denotes the weights of the policy network. **p** represents the “drop” likelihood (**p**_*n*_ ∈ [0, 1]), and is the policy network's output after sigmoid activation. The action vector **a** is determined by **p**, where **a**_*n*_ = 0 refers to drop the *n*-th block, otherwise keeping the corresponding block.

#### 2.2.2. Reward Function

3D Segmentation is generally considered to be voxel-level classification, thus every voxel has to receive evaluation feedback for the corresponding action. Thus, we design the following voxel-level reward function:

(2) $$R(a)=\left\{ \begin{array}{l} 1-{\left(\frac{|a{|}_{0}}{N}\right)}^{2}\;if\;VDC(i)=\frac{1}{2}\\ \;-\tau \;if\;VDC(i)=1, \end{array}\right.$$

where ${\left(\frac{|a{|}_{0}}{N}\right)}^{2}$ calculates the block usage. When the prediction of a voxel *i* is the same as the label, we encourage dropping more blocks by giving a larger reward to the policy. On the other hand, we penalize using τ, which balances block usage and segmentation accuracy. When τ is a large value, the policy is prone to have a more solid segmentation result; otherwise, it is more likely to drop blocks. We designed Voxel Dice Coefficient (VDC) to identify which pixel should be penalized:

(3) $$\begin{array}{l} VDC\left(i\right)=\frac{2*\left(y_{i}^{\prime }⋂y_{i}\right)+1}{y_{i}^{\prime }+y_{i}+1}, \end{array}$$

where *y*_*i*_ ∈ {0, 1} and $y_{i}^{\prime }\in \left\{0,1\right\}$ denotes the ground truth and prediction for voxel *i*, respectively.

#### 2.2.3. Learning Strategy

Finally, we maximize the expected reward E_**a**~π__**W**_[*R*(**a**)] to train the policy network. We employ policy gradient to calculate the gradient of the expected reward:

(4) $${▽}_{W}E_{a\sim \pi_{W}}[R(a)]=E[R(a){▽}_{W}\log\;\pi_{W}(a|x)]$$

(5) $$=E[R(a){▽}_{W}\log\;\prod_{n=1}^{N}p_{n}^{a_{n}}{\left(1-p_{n}\right)}^{1-a_{n}}]$$

(6) $$=E[R(a){▽}_{W}\sum_{n=1}^{N}\log[p_{n}a_{n}+(1-p_{n})(1-a_{n})]].$$

The Equation (6) can be approximated by Monte-Carlo sampling strategy.

To achieve efficient training, we further employ curriculum learning (Bengio et al., 2009) to train the policy network. Specifically, for epoch *c* (*c* < *N*), the first *N* − *c* blocks are kept, while the learning is conduct on the last *c* blocks. As *c* increases, more and more blocks join the optimization until all blocks are involved. Algorithm 1 shows the training procedure of the proposed network.

**Algorithm 1:**
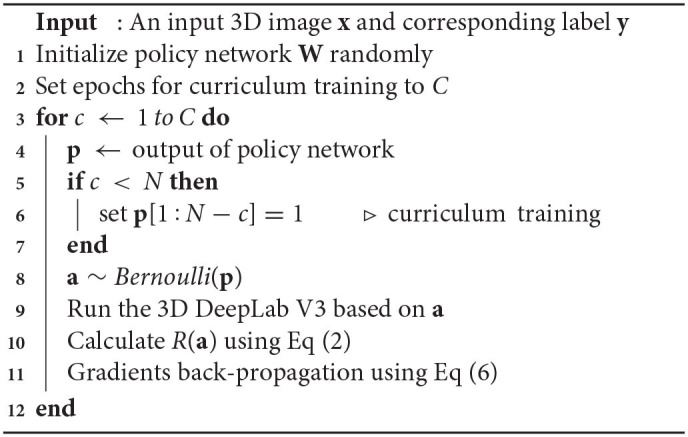
**Training of the policy network**.

## 3. Experiments and Results

### 3.1. Materials and Implementation Details

#### 3.1.1. Materials

Experiments were carried on liver CT images from LiTS challenge (Bilic et al., 2019). LiTS dataset contains 131 contrast-enhanced CT images acquired from six clinical centers around the world. 3DIRCADb is a subset from the LiTS dataset with 22 cases. Our network was trained using 109 cases from LiTS without data from 3DIRCADb, and then evaluated on the 3DIRCADb subset using Dice metric.

#### 3.1.2. Implementation Details

The experiments were conducted using 3D DeepLab V3 network with 18-layers and 50-layers, respectively. We adopted Adam (Kingma and Ba, 2014) with learning rate of 0.01 and batch size of 4 and 11 for ResNet-18 and ResNet-50, respectively. In addition, we utilized learning rate scheduler that decreases by 0.1 for every 100 epochs. The maximum epoch was set to 400.

For the policy network, we set learning rate to 0.001, τ to 50 and used the batch size of 1 and 5 for ResNet-18 and ResNet-50, respectively.

### 3.2. Performance Evaluation

#### 3.2.1. Investigation of Blocks' Dependencies

**Figure 3:**
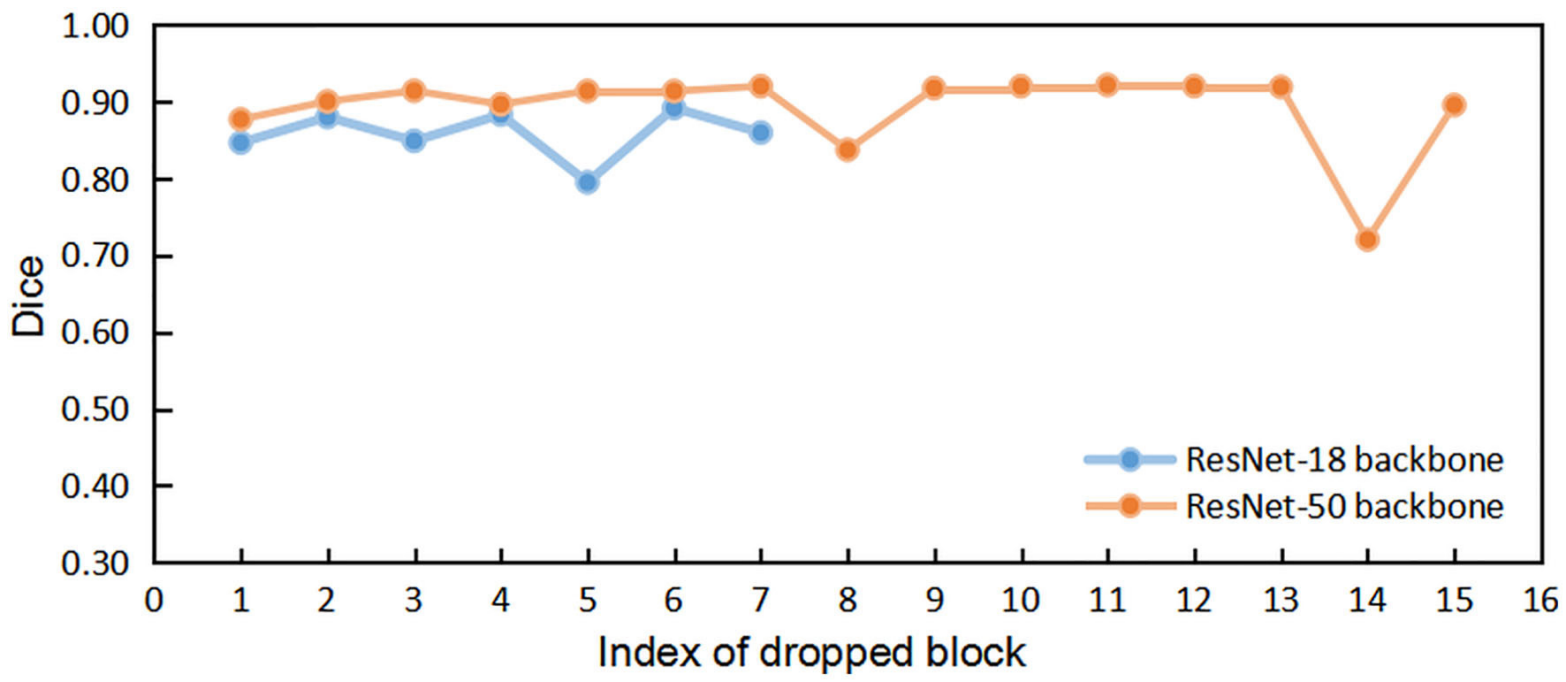
The comparisons of segmentation performance from dropping different individual block of residual backbones.

We first implemented the DropN strategy, which means dropping a single *n*-th block in residual backbone, to observe the dependencies of different blocks. We executed this strategy to ResNet-18 and ResNet-50 backbone which has 8 and 16 residual blocks, respectively. Figure 3 shows that dropping individual block from residual backbone has a minimal impact on Dice evaluation except for few blocks. This suggests that different blocks in ResNet backbone do not share strong dependencies and most blocks are not indispensable for the accurate segmentation. Thus, dropping blocks in inference is feasible for segmentation.

#### 3.2.2. Heuristic Dropping Strategies

We then evaluated three manual dropping strategies as follows:

1. DropFirstN, which means to drop all blocks before the *n*-th block;
2. DropLastN, which means to drop all blocks after the *n*-th block;
3. DropRandomN, which means to drop *n* blocks randomly.

Note that DropRandomN is a random strategy, thus for each *n* we performed 100 and 500 repeated experiments for ResNet-18 and ResNet-50, respectively.

**Figure 4:**
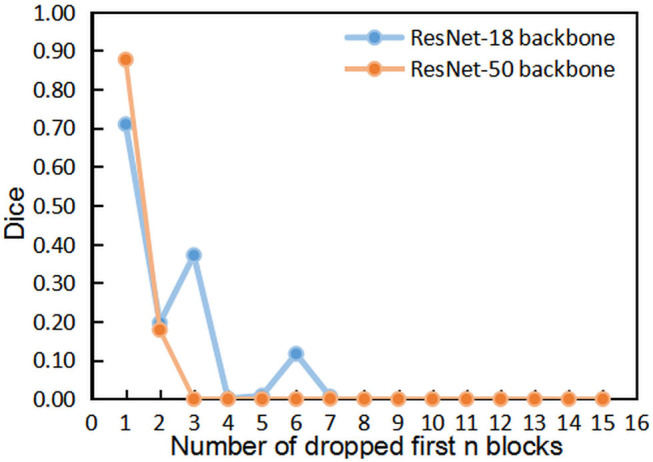
The comparisons of segmentation performance from dropping the first *n* blocks of residual backbones.

**Figure 5:**
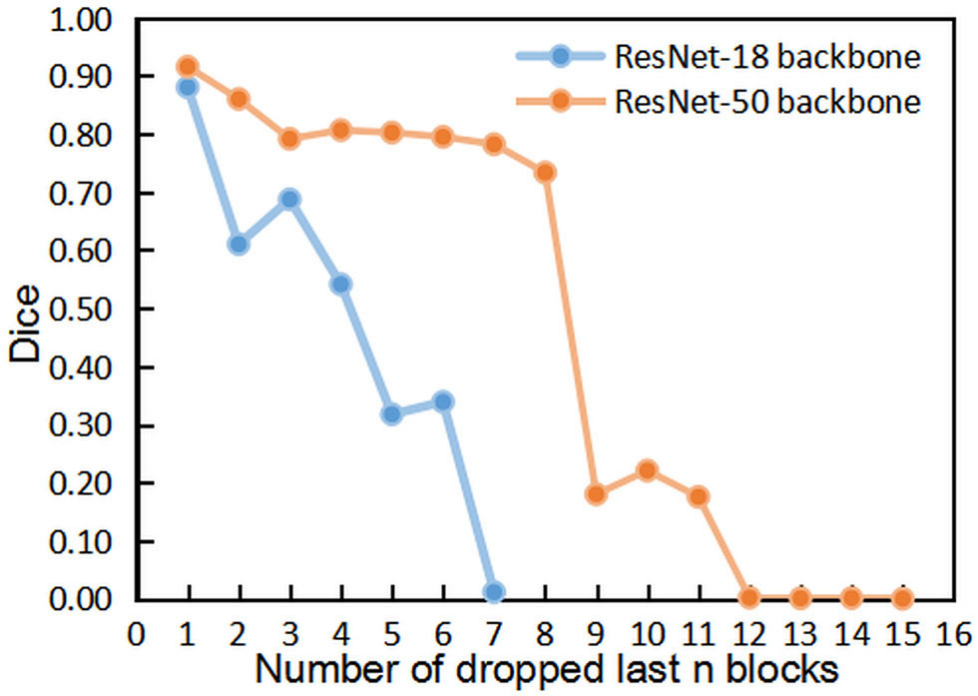
The comparisons of segmentation performance from dropping the last *n* blocks of residual backbones.

Figures 4, 5 show the results of DropFirstN and DropLastN, respectively. It can be observed from Figure 4 that first several blocks were relatively important for ResNet backbone. The Dice value dropped to almost 0 when the first three blocks were dropped. As shown in Figure 5, for ResNet-50, dropping the last 8 blocks didn't affect segmentation performance sharply.

**Figure 6:**
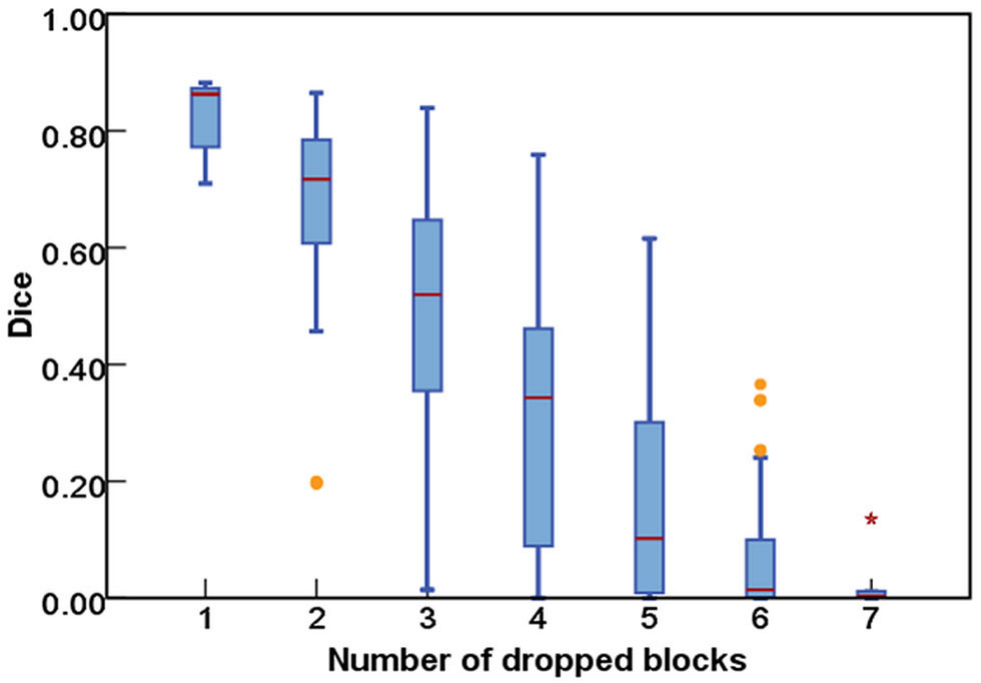
The comparisons of segmentation performance from randomly dropping *n* blocks of ResNet-18.

**Figure 7:**
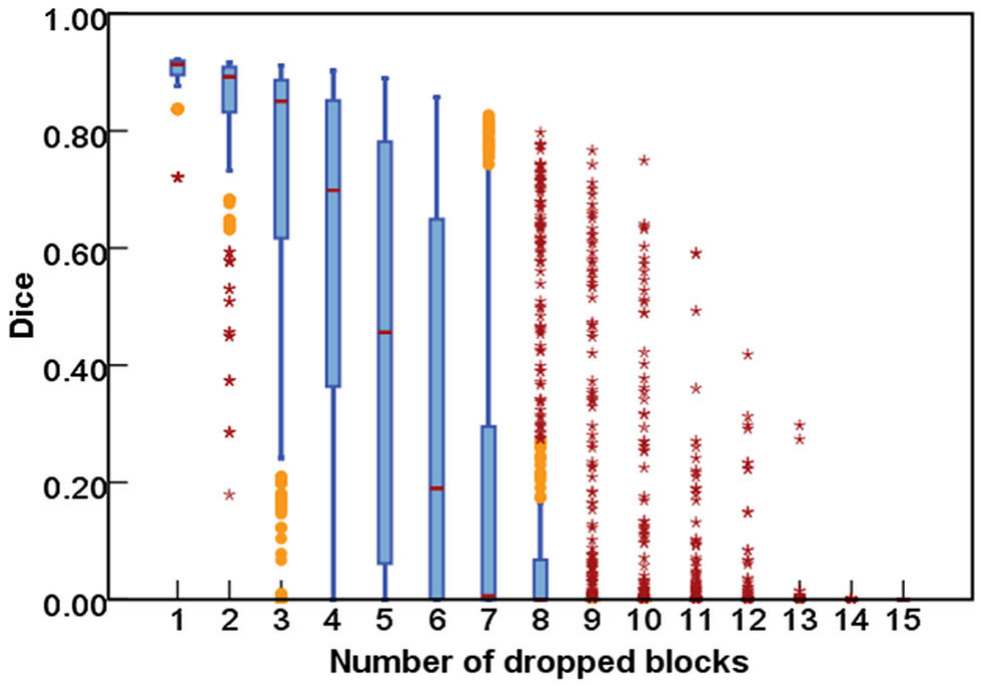
The comparisons of segmentation performance from randomly dropping *n* blocks of ResNet-50.

As for the DropRandomN, with the increase of dropped blocks in the shallow backbone, the segmentation performance gradually decreased, as seen in Figure 6. In contrast, for the ResNet-50, the average Dice was almost 0 when 8 blocks were randomly dropped, as shown in Figure 7.

#### 3.2.3. *AutoPath*

We compared the segmentation performance of heuristic strategies (i.e., DropFirstN, DropLastN, DropRandomN) and the proposed *AutoPath* at the same dropping level. Table 1 reports that by considering image-specific input, our *AutoPath* can achieve an average block dropping ratio of more than 25%, meanwhile with only 2 and 4% decrease of Dice values for ResNet-18 and ResNet-50, respectively. Also can be seen from Table 1 that by dropping the same number of residual blocks, our *AutoPath* outperformed other heuristic strategies by a large margin.

**Table 1:** The comparisons of segmentation performance from heuristic strategies and *AutoPath* at the same dropping level.

	**ResNet-18 backbone**		**ResNet-50 backbone**	
	**Dice**	**Dropped blocks**	**Dice**	**Dropped blocks**
DropFirstN	19.67%	2	0.00%	4
DropLastN	61.03%	2	80.66%	4
DropRandomN	67.80%	2	59.11%	4
Ours	87.11%	2.4	89.14%	4.5
Full backbone	89.54%	8 used	93.16%	16 used

**Figure 8:**
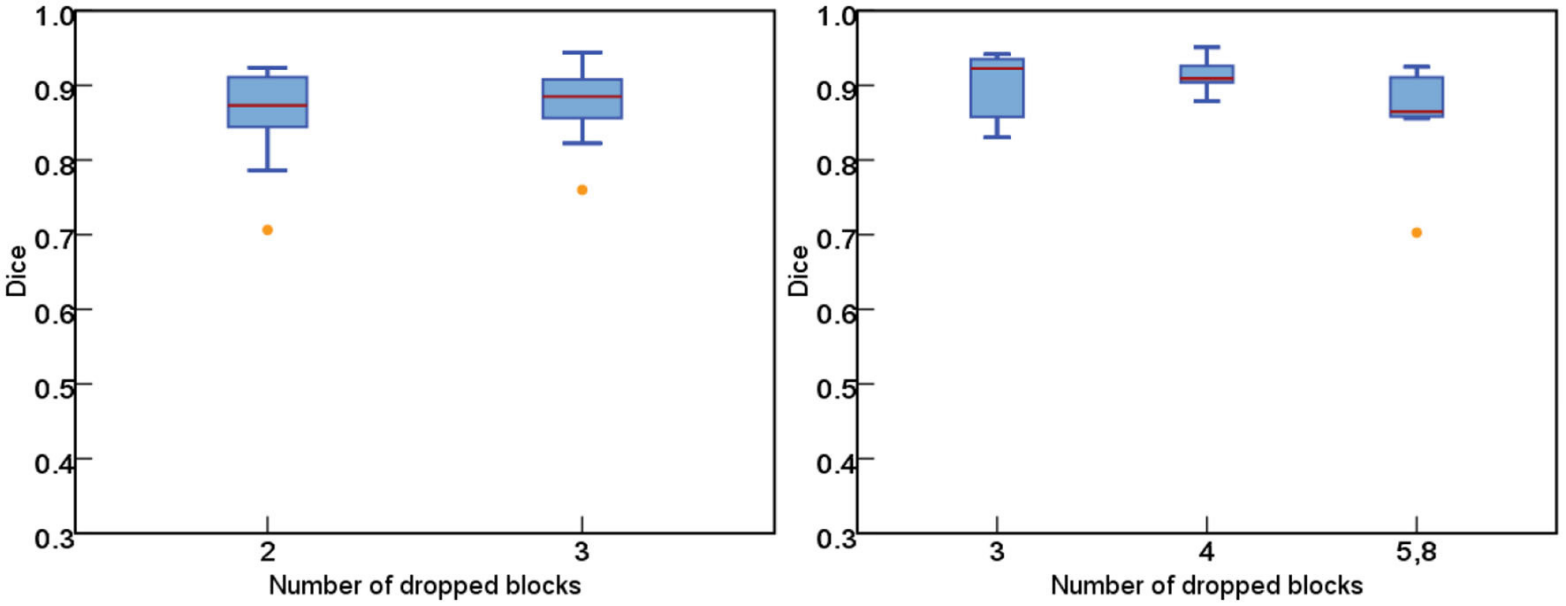
The statistics of the *AutoPath* for all testing data. For most cases, *AutoPath* can provide high quality segmentation with fewer block usage. Left: ResNet-18; Right: ResNet-50.

**Figure 9:**
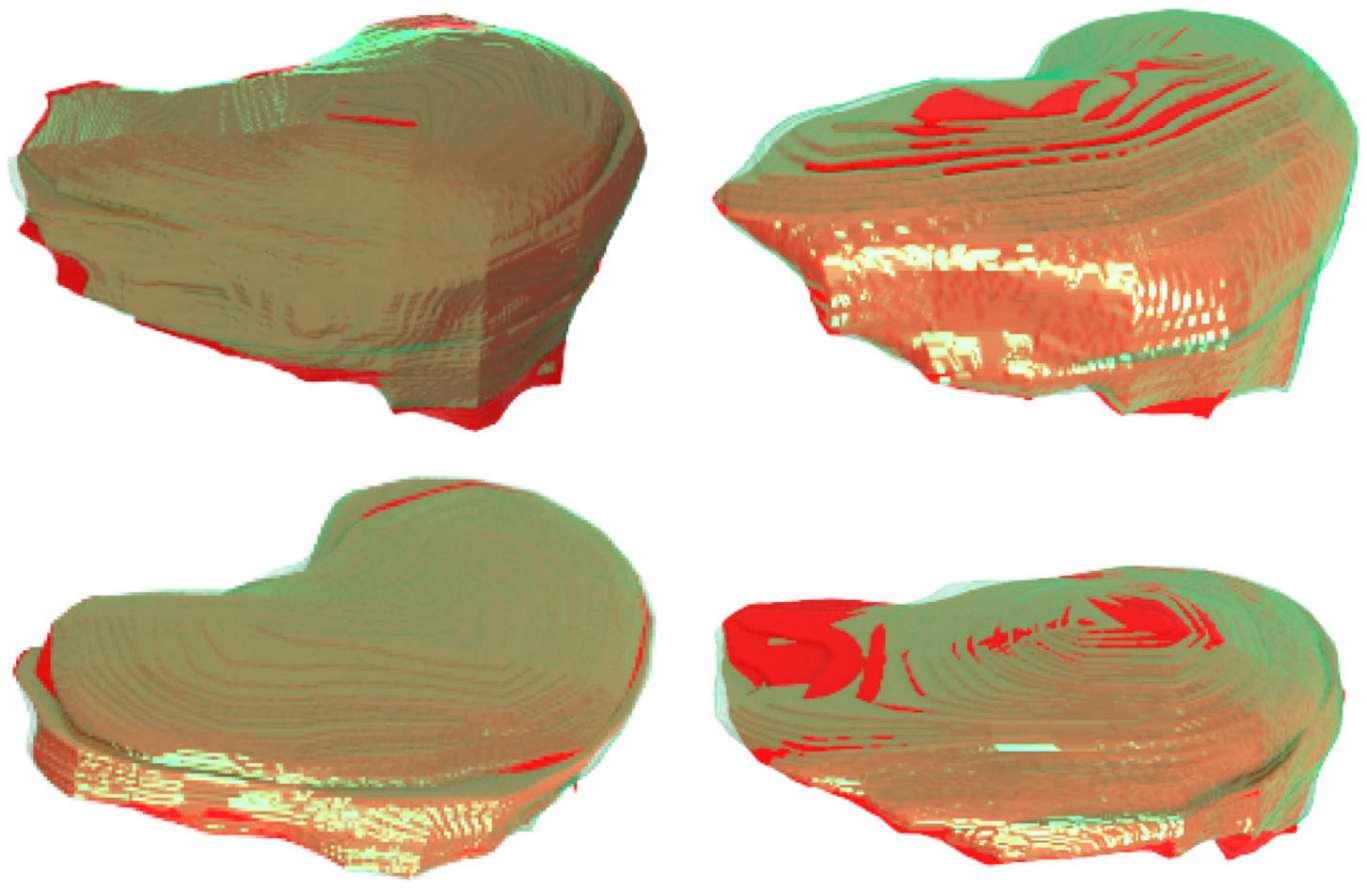
3D visualizations of some segmentation results obtained using *AutoPath* (green) and full backbone (red), respectively. It can be observed that the segmentation performance from the proposed *AutoPath* was comparable to that of the full backbone architecture.

**Figure 10:**
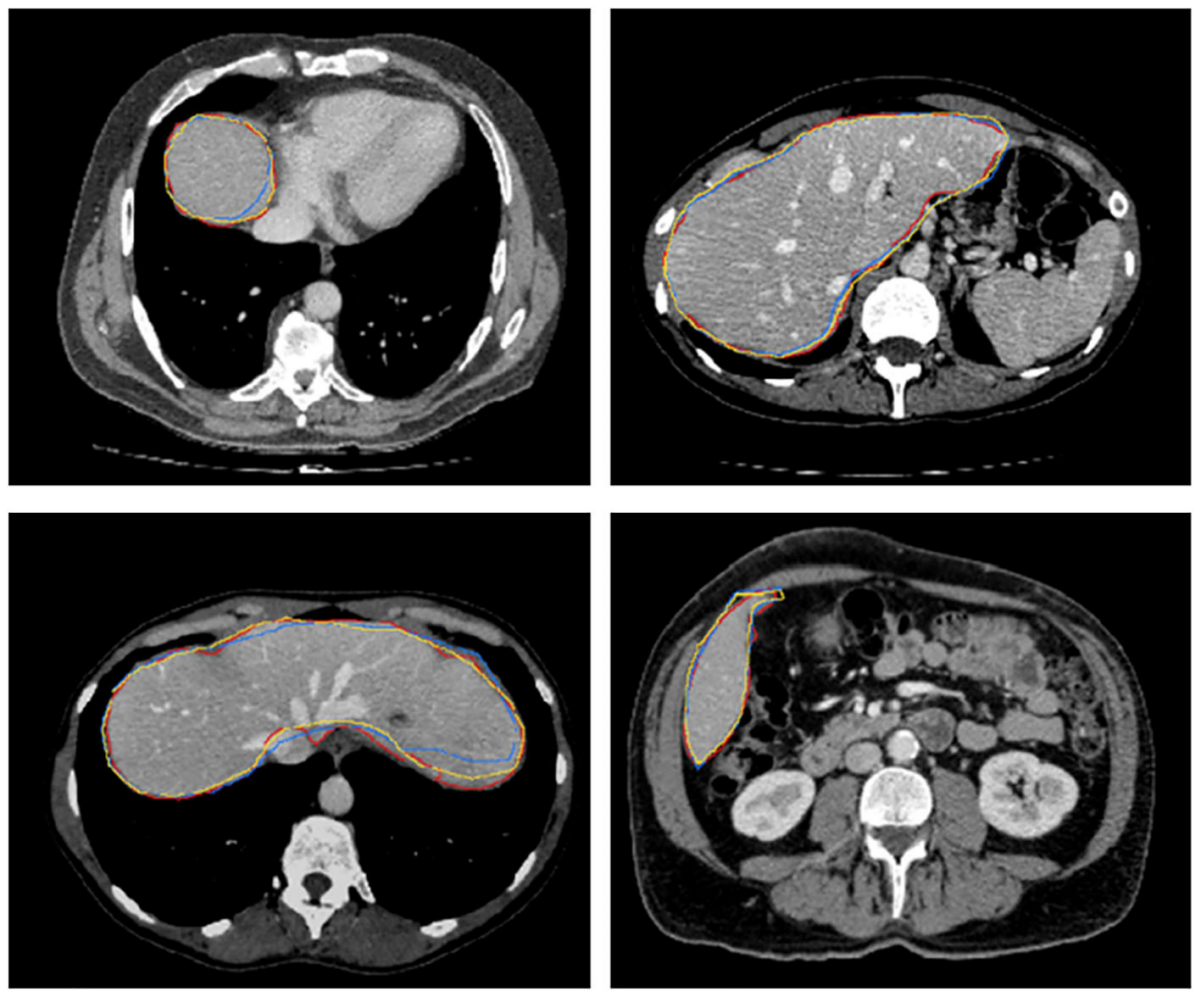
2D slices of some segmentation results obtained using *AutoPath* (yellow), full backbone (blue), and the ground truth (red), respectively.

Figure 8 further plots the statistics of the *AutoPath* for all testing data. For the image-specific inference, the *AutoPath* selectively dropped 2 or 3 blocks for ResNet-18, and 3, 4, 5, or 8 blocks for ResNet-50. For most cases, *AutoPath* can maintain a high quality segmentation with fewer block usage, which demonstrates its promising application for real clinical circumstance. Figures 9, 10 visualizes some 3D and 2D segmentation results obtained using *AutoPath* and full backbone, respectively. It can be observed that the segmentation performance from the proposed *AutoPath* was comparable to that of the full backbone architecture.

## 4. Discussion and Conclusion

This paper develops a reinforcement learning method to select image-specific and efficient inference paths for 3D segmentation, which addresses the problem of huge computational burden for 3D segmentation networks. To our best knowledge, this is the first study of the dynamic inference path for 3D segmentation. We refer to it as *AutoPath*, which can leverage an image-specific path including fewer residual blocks to attain accurate prediction. To achieve this, we train a network to determine the policy of block dropping and the pretrained segmentation network executes inference according to this policy. We conducted extensive experiments on the liver CT dataset using 3D DeepLab V3 network with 18-layers and 50-layers, respectively. Experimental results demonstrate that *AutoPath* is a reliable method for the dynamic inference in 3D segmentation.

Deep neural networks offer excellent segmentation performance, yet their computational expense restrict their clinical usage, especially for the 3D segmentation. To tackle this issue, various compressed models have been proposed (Li et al., 2016; He et al., 2017, 2018; Liu et al., 2017; Luo et al., 2017). While the network efficiency has been improved somehow, the solution is a one-size-fit-all network that omits different inputs' complexity. In contrast, we investigate adaptively allocating computation across a CNN model according to specific input. Furthermore, our image-specific inference is conducted on the trained network, thus do not have to spend extra time for the network retuning.

In this study, although the DeepLabV3 network was employed as backbone to equip with residual structures, it could be replaced using other backbone architectures. With regard to our image-specific inference method, the residual structures are the most crucial components but not the design of backbones. In medical segmentation tasks, various CNN-based approaches have employed residual structures. For example, the encoder of HD-Net (Jia et al., 2019) is based on 3D ResNet-101 and BOWDA-Net (Zhu et al., 2019) utilizes dense connection multiple times. In addition, Xiao et al. (2018) propose a weighted Res-UNet which replaces the convolution block with residual block to achieve remarkable results in retina vessel segmentation. Furthermore, there are some improved structures based on ResNet, such as ResNext (Xie et al., 2017), SE-Net (Hu et al., 2018), and SK-Net (Li et al., 2019). Our proposed method can be utilized to dynamically distribute computation across their residual blocks for image-specific segmentation inference.

Our current scheme mainly focuses on the usage reduction of the residual blocks due to their independent design. It may not be directly adopted to other network configurations without residual structures. Future work may further investigate the dynamic inference for other network configurations. In addition, although our method attained satisfactory performance on liver CT volumes, further validations on large amount of various medical images will be conducted to investigate the robustness and generalization ability of the proposed scheme.

## Data Availability Statement

Publicly available datasets were analyzed in this study. This data can be found here: https://chaos.grand-challenge.org/Combined_Healthy_Abdominal_Organ_Segmentation.

## Author Contributions

DS, YW, DN, and TW response for study design. DS implemented the research. DS and YW conceived the experiments. DS conducted the experiments. DS, YW, and TW analyzed the results. DS and YW wrote the main manuscript text and prepared the figures. All authors reviewed the manuscript.

## Conflict of Interest

The authors declare that the research was conducted in the absence of any commercial or financial relationships that could be construed as a potential conflict of interest.
